# CD8+ T Cells Responding to the Middle East Respiratory Syndrome Coronavirus Nucleocapsid Protein Delivered by Vaccinia Virus MVA in Mice

**DOI:** 10.3390/v10120718

**Published:** 2018-12-16

**Authors:** Svenja Veit, Sylvia Jany, Robert Fux, Gerd Sutter, Asisa Volz

**Affiliations:** 1 Institute for Infectious Diseases and Zoonoses, LMU Munich, 80539 Munich, Germany; svenja.veit@micro.vetmed.uni-muenchen.de (S.V.); sylvia.jany@micro.vetmed.uni-muenchen.de (S.J.); robert.fux@micro.vetmed.uni-muenchen.de (R.F.); asisa.volz@micro.vetmed.uni-muenchen.de (A.V.); 2 German Center for Infection Research (DZIF), partner site Munich, 80539 Munich, Germany

**Keywords:** MERS-CoV, MERS-CoV nucleocapsid protein, murine CD8+ T cell epitope, MVA vaccine

## Abstract

Middle East respiratory syndrome coronavirus (MERS-CoV), a novel infectious agent causing severe respiratory disease and death in humans, was first described in 2012. Antibodies directed against the MERS-CoV spike (S) protein are thought to play a major role in controlling MERS-CoV infection and in mediating vaccine-induced protective immunity. In contrast, relatively little is known about the role of T cell responses and the antigenic targets of MERS-CoV that are recognized by CD8+ T cells. In this study, the highly conserved MERS-CoV nucleocapsid (N) protein served as a target immunogen to elicit MERS-CoV-specific cellular immune responses. Modified Vaccinia virus Ankara (MVA), a safety-tested strain of vaccinia virus for preclinical and clinical vaccine research, was used for generating MVA-MERS-N expressing recombinant N protein. Overlapping peptides spanning the whole MERS-CoV N polypeptide were used to identify major histocompatibility complex class I/II-restricted T cell responses in BALB/c mice immunized with MVA-MERS-N. We have identified a H2-d restricted decamer peptide epitope in the MERS-N protein with CD8+ T cell antigenicity. The identification of this epitope, and the availability of the MVA-MERS-N candidate vaccine, will help to evaluate MERS-N-specific immune responses and the potential immune correlates of vaccine-mediated protection in the appropriate murine models of MERS-CoV infection.

## 1. Introduction

The Middle East respiratory syndrome coronavirus (MERS-CoV), a hitherto unknown β-coronavirus, emerged as a causative agent of a severe respiratory disease in humans in 2012. This new coronavirus was first isolated from the sputum of a patient suffering from severe pneumonia and renal failure [1]. To date, the MERS-CoV still causes disease and death in humans with a total of 2260 confirmed cases including 803 fatalities [2,3]. Epidemiological data suggest that the MERS-CoV is endemic in Saudi Arabia, which accounts for the majority of primary community-acquired cases. Many of those primary cases are due to virus exposure through direct contact with dromedary camels, the primary animal reservoir of MERS-CoV. Alternatively, camel workers undergoing subclinical infections are suggested to mediate virus transmission to other susceptible individuals [4,5]. Other outbreaks of MERS have been caused by nosocomial transmissions in health care settings [6,7,8,9]. Most of the MERS-CoV infections occur within the Arabian Peninsula, i.e., Saudi Arabia, Qatar, and United Arab Emirates, however MERS cases have been reported in various other countries around the world [9,10]. At present, there is still relatively little known about the pathogenesis of MERS-CoV. The highest incidence of severe disease is observed in elderly and immunocompromised individuals [11]. Those at general risk for infections are health care workers and people in close contact with dromedary camels [12,13]. These groups are therefore considered relevant target populations for prophylactic vaccination against MERS-CoV infection and prevention of MERS. The World Health Organization (WHO) and the Coalition for Epidemic Preparedness Innovations (CEPI) have listed MERS as priority target for vaccine development [14]. However, so far, no candidate vaccines have proceeded beyond phase I/IIa clinical testing [15]. One of these candidate vaccines is based on Modified Vaccinia virus Ankara (MVA), a safety-tested and replication-deficient vaccinia virus serving as an advanced viral vector platform for the development of new vaccines against infectious diseases and cancer (for review see [16]). In that context, we still know relatively little about the correlates of vaccine induced protection against MERS-CoV. It is well-known that virus-neutralizing antibodies directed against the spike glycoprotein (S protein) correlate with protective immunity against coronavirus infections in general [17,18,19,20]. Since the S protein is present on the cell surface, S protein is considered as the major antigen to induce virus neutralizing antibodies and as a key immunogen for the development of MERS-CoV candidate vaccines [21,22,23,24,25]. However, based on current knowledge from the biology of β-coronaviruses, we hypothesize that also other viral proteins warrant consideration as immunogens and targets of virus-specific antibodies and T cells. Among those, the nucleocapsid protein (N protein) is produced at high levels in infected cells and has been proposed as useful candidate protein for clinical diagnosis [26,27,28,29,30]. The coronavirus N proteins have been associated with multiple functions in the virus life cycle including the regulation of viral RNA synthesis, the packaging of the viral RNA in helical nucleocapsids, and in virion assembly through interaction with the viral M protein [31,32,33,34]. Furthermore, several reports suggest that the severe acute respiratory syndrome coronavirus (SARS-CoV) N protein functions as an immune evasion protein and an antagonist of the host interferon response [35,36,37]. Recently, the overexpression of MERS-CoV N in human A549 cells was found to be linked to an up-regulation of antiviral host gene expression including the synthesis of the inflammatory chemokine CXCL10 [38]. Despite this possible immune modulatory activity, SARS-CoV N-specific immune responses are reported to be long-lived and more broadly reactive when compared to SARS-CoV S-specific immunity [39]. Likewise, we were curious as to the suitability of the MERS-CoV N protein to serve as a vaccine antigen. The N protein is not present on the surface of MERS-CoV particles nor is it predicted to be expressed on the surface membrane of MVA infected cells. From this we hypothesized that the most relevant part of MERS-CoV N-specific immunity is based on CD8+ T cell responses relying on the processing and presentation of intracellular antigens. Currently, there is little information about MERS-CoV N-specific immune responses including the in vivo induction of N-specific cellular immunity.

In this study, we investigated the synthesis and delivery of the MERS-CoV N protein as a privileged antigen by a MVA vector virus. The recombinant MVA expressing a synthetic gene sequence of full-length MERS-CoV N (MVA-MERS-N) proved genetically stable and fully replication-competent in chicken embryo fibroblasts, an established cell substrate for MVA vaccine manufacturing. Upon in vitro infection, MVA-MERS-N produced high amounts of the heterologous protein that were detectable with MERS-CoV N-specific antibodies. Furthermore, MVA-MERS-N was tested as an experimental vaccine in BALB/c mice and elicited MERS-CoV N-specific interferon γ (IFN-γ)-producing CD8+ T cells. Using peptide library covering the whole MERS-CoV N polypeptide, we identified new H2-d restricted peptide epitopes of MERS-CoV N in BALB/c mice. This data will be highly relevant for further assessment of N antigen-specific immune responses in the well-established MERS-CoV-BALB/c mouse immunization/challenge model [40,41,42,43,44].

## 2. Materials and Methods

### 2.1. Mice

Female BALB/c mice (6 to 10-week-old) were purchased from Charles River Laboratories (Sulzfeld, Germany). For experimental work, mice were housed in an isolated (ISO) cage unit (Tecniplast, Hohenpeißenberg, Germany) and had free access to food and water. All animal experiments were handled in compliance with the German regulations for animal experimentation (Animal Welfare Act, approved by the Government of Upper Bavaria, Munich, Germany).

### 2.2. Cells

Primary chicken embryo fibroblasts (CEF) were prepared from 10-day-old chicken embryos (SPF eggs, VALO, Cuxhaven, Germany) and maintained in Minimum Essential Medium Eagle (MEM) (SIGMA-ALDRICH, Taufkirchen, Germany) containing 10% heat-inactivated fetal bovine serum (FBS) (SIGMA-ALDRICH, Taufkirchen, Germany), 1% Penicillin-Streptomycin (SIGMA-ALDRICH, Taufkirchen, Germany), and 1% MEM non-essential amino acid solution (SIGMA-ALDRICH, Taufkirchen, Germany). Human HeLa (ATCC CCL-2) cells were maintained in MEM containing 10% FBS and 1% Penicillin-Streptomycin. Human HaCat (CLS Cell Lines Service GmbH, Eppelheim, Germany) cells were cultured in Dulbecco`s Modified Eagle`s Medium (SIGMA-ALDRICH, Taufkirchen, Germany) supplemented with 10% heat-inactivated FBS, 2% HEPES-solution (SIGMA-ALDRICH, Taufkirchen, Germany) and antibiotics as described above. All cells were maintained at 37 °C and 5% CO_2_ atmosphere.

### 2.3. Plasmid Constructions

The cDNA encoding the entire amino acid (aa) sequence (413 aa) of the MERS-CoV N protein was in silico modified by introducing silent codon alterations to remove three termination signals (TTTTTNT) for vaccinia virus early transcription and two G/C nucleotide runs from the original MERS-CoV gene sequence (Human betacoronavirus 2c EMC/2012, GenBank accession no. JX869059). A cDNA fragment was generated by DNA synthesis (Invitrogen Life Technology, Regensburg, Germany) and cloned into the MVA transfer plasmid pIIIH5red [45] to place the MERS-CoV N gene sequence under the transcriptional control of the vaccinia virus early/late promoter PmH5 [46] resulting in the MVA vector plasmid pIIIH5red-MERS-N.

### 2.4. Generation of Recombinant Virus

Recombinant MVA was generated using standard methodology as described previously [45]. Briefly, monolayers of nearly confluent CEF grown in six-well tissue culture plates (Sarstedt, Nürnbrecht, Germany) were infected with non-recombinant MVA (clonal isolate MVA F6) at 0.05 multiplicity of infection (MOI) and, 45 min after infection, CEF cells were transfected with plasmid pIIIH5red-MERS-N DNA using X-tremeGENE DNA Transfection Reagent Lipofectamine (Roche Diagnostics, Penzberg, Germany) as recommended by the manufacturer. At 48 h after infection, the cell cultures were harvested and recombinant MVA expressing the MERS-CoV N protein was clonally isolated by consecutive rounds of plaque purification in CEF by screening for transient co-expression of the red fluorescent marker protein mCherry.

Quality control experiments were performed using standard methodology [45]. Genetic identity and genetic stability of the recombinant virus were assessed via polymerase chain reaction (PCR) analysis of the genomic viral DNA. Replicative capacities of the MVA vector virus was tested in multi-step growth experiments in CEF and human HaCat and HeLa cells.

To generate high titer vaccine preparations for preclinical studies, recombinant MVA was amplified in CEF, purified by ultracentrifugation through 36% sucrose cushions, resuspended in 10 mM Tris-HCl buffer, pH 9.0, and stored at −80 °C. The sucrose purified MVA-MERS-N vaccine preparations corresponded in total protein/total DNA content to the purity profile of a MVA candidate vaccine for human use.

### 2.5. Western Blot Analysis of Recombinant Proteins

Confluent cell monolayers of CEF or HaCat cells were infected at a MOI of 5 with recombinant MVA expressing the MERS-CoV N or S protein [23]. Non-infected (mock) or wild-type MVA-infected cells served as controls. Cell lysates were prepared at different time points after infection and stored at −80 °C. Total cell proteins were resolved by electrophoresis in a sodium dodecyl sulfate (SDS)-10% polyacrylamide gel (SDS-PAGE) and subsequently transferred onto a nitrocellulose membrane via electroblotting. After 1 h blocking in a phosphate buffered saline (PBS) buffer containing 1% (w/v) non-fat dried milk and 0.1% (v/v) NP-40 detergent, the blots were incubated with monoclonal mouse anti-MERS-CoV Nucleocapsid antibody (Sino Biological, Beijing, China, 1:1000), monoclonal rabbit anti-MERS-CoV Spike Protein S1 Antibody (Sino Biological, 1:500), or polyclonal sera from MERS-CoV infected rabbits or cynomolgus macaques (kindly provided by Dr. Bart Haagmans, Erasmus Medical Center, Rotterdam, 1:1000) [23] as primary antibodies. After washing with 0.1% NP-40 in PBS, the blots were incubated with anti-mouse IgG (1:5000), or anti-rabbit IgG antibody (1:5000), or protein A (1:1000) conjugated to horseradish peroxidase (Cell Signaling Technology, Frankfurt am Main, Germany). After further washing, blots were developed using SuperSignal® West Dura Extended Duration substrate (Thermo Fisher Scientific, Planegg, Gemany).

### 2.6. Immunization Experiments in Mice

Groups of female BALB/c mice (*n* = 2 to 5) were immunized twice within a 21-day interval with 10^8^ plaque-forming-units (PFU) of recombinant MVA-MERS-N or non-recombinant MVA (MVA) or PBS as mock vaccine. Vaccinations were given via the intramuscular (i.m.) or intraperitoneal (i.p.) route using 25 µL (i.m.) or 200 µL (i.p.) volumes per inoculation. All mice were monitored daily for welfare and potential adverse events of immunization. At day 8 post prime-boost immunization, animals were sacrificed by cervical dislocation and spleens were taken for T cell analysis.

### 2.7. Synthetic Peptides and Design of Peptide Pools

For T cell immune monitoring, we identified 101 individual synthetic peptides (assigned as 1 to 101) in silico spanning the entire MERS-CoV N protein sequence (Human betacoronavirus 2c EMC/2012, GenBank accession no. JX869059). This peptide library was designed to contain 15-mer peptides overlapping by 11 aa. Eighty-four peptides could be synthesized (Thermo Fisher Scientific) and were organized into two-dimensional matrix peptide pools (V1 to V9 and H1 to H9) containing 9 or 10 peptides as described previously [47,48]. For further T cell epitope mapping, the 11 aa sequence shared between peptide #89 and #90 was trimmed into 8-10-mer peptides, which were also obtained from Thermo Fisher Scientific. All peptides were dissolved in PBS to a concentration of 2 mg/mL and stored at −20 °C until use.

### 2.8. T cell Analysis by Enzyme-Linked Immunospot (ELISPOT)

Spleens were harvested on day 8 post prime-boost vaccination. Splenocytes were prepared by passing through a 70 µm strainer (Falcon®, A Corning Brand, Corning, USA) and incubating with Red Blood Cell Lysis Buffer (SIGMA-ALDRICH, Taufkirchen, Germany). Cells were washed and resuspended in RPMI 1640 medium (SIGMA-ALDRICH) containing 10% heat inactivated FBS and 1% Penicillin-Streptomycin. Splenocytes were further processed by using the QuadroMACS Kit (Miltenyi Biotec, Bergisch Gladbach, Germany) to separate CD8+ and CD4+ splenocytes with MACS Micro Beads (Miltenyi Biotec, Bergisch Gladbach, Germany).

IFN-γ-producing T cells were measured using ELISPOT assays (Elispot kit for mouse IFN-γ, MABTECH, Germany) following the manufacturer`s instructions. Briefly, 1 × 10^6^ splenocytes were seeded in 96-well plates (Sarstedt, Nürnbrecht, Germany) and stimulated with peptide pools or individual peptides (2 µg peptide/mL RPMI 1640 medium) at 37 °C for 48 h. Non-stimulated cells and cells treated with phorbol myristate acetate (PMA) (SIGMA-ALDRICH) and ionomycin (SIGMA-ALDRICH, Taufkirchen, Germany) or MVA F2L_26-34_ peptide (F2L, SPGAAGYDL, Thermo Fisher Scientific, Planegg, Germany) [49] served as negative and positive controls, respectively. Automated ELISPOT plate reader software (A.EL.VIS Eli.Scan, A.EL.VIS ELISPOT Analysis Software, Hannover, Germany) was used to count and analyze spots.

### 2.9. T Cell Analysis by Intracellular Cytokine Staining (ICS) and Flow Cytometry

Splenocytes were prepared as described above. Splenocytes were added to 96-well plates (1 × 10⁶ cells/well) and stimulated for 6 h with MERS-CoV N-specific peptide (at 8 µg peptide/mL RPMI 1640 medium) in presence of the protein transport inhibitor Brefeldin A (Biolegend, San Diego, CA, USA; 5 µg/mL). Non-stimulated cells served as a background control and cells stimulated with 5 ng/mL PMA and 500 ng/mL ionomycin or with F2L peptide (8 µg/mL RPMI 1640 medium) were used as positive controls. After stimulation, cell surface antigens were stained using PE-conjugated anti-mouse CD3 (clone: 17A2, Biolegend, San Diego, CA, USA), PE/Cy7-conjugated anti-mouse CD4 (clone: GK1.5, Biolegend, San Diego, CA, USA), or FITC-conjugated anti-mouse CD8a (clone: 5H10-1, Biolegend, San Diego, CA, USA) antibody and incubated for 30 min on ice. The surface-stained cells were washed with staining buffer (MACS QuantTM Running Buffer, Miltenyi Biotec), then fixed and permeabilized with Fixation- and Perm/Wash-Buffer (Biolegend, San Diego, CA, USA), and finally stained for intracellular IFN-γ expression using APC-conjugated anti-mouse-IFN-γ antibody (clone: XMG1.2, Biolegend, San Diego, CA, USA) for 30 min on ice. Following final washes, cells were resuspended in staining buffer and analyzed using the MACS Quant® VYB flow cytometer (Miltenyi Biotec, Bergisch Gladbach, Germany).

### 2.10. Statistical Analysis

Statistical analysis was performed by t-test using GraphPad Prism version 5 software (GraphPad software, San Diego CA, USA); P-values less than 0.05 were considered to be statistically significant.

## 3. Results

### 3.1. Construction and Characterization of Recombinant MVA Expressing MERS-CoV N Gene Encoding Sequences

**Figure 1:**
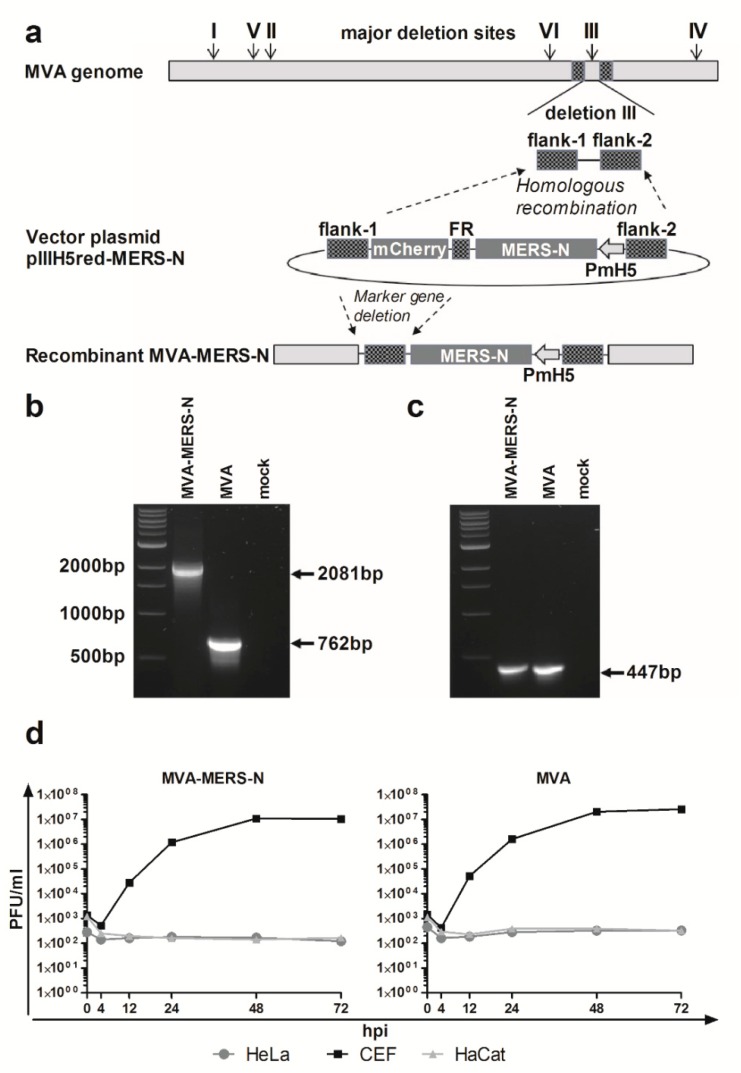
Generation and characterization of recombinant Modified Vaccinia virus Ankara expressing the Middle East respiratory syndrome coronavirus N protein (MVA-MERS-N); (**a**) Schematic diagram of the MVA genome indicating the major deletion sites I-VI on the top. Flank-1 and flank-2 refer to MVA DNA sequences adjacent to corresponding insertion site. Deletion III was used to insert MERS-N encoding gene sequences under the transcriptional control of the vaccinia virus promoter PmH5. Repetitive sequences (FR) were designed to remove the mCherry marker by intragenomic homologous recombination (marker gene deletion); (**b**,**c**) PCR analyses of genomic viral DNA using oligonucleotide primers to confirm the correct insertion of recombinant MERS-N gene into deletion III (**b**), and the genetic integrity of the MVA genome for the C7L gene locus (**c**); (**d**) Multi-step growth analysis of recombinant MVA-MERS-N and non-recombinant MVA (MVA); Chicken embryo fibroblasts (CEF) and human HaCat or HeLa cells were infected at a multiplicity of infection (MOI) of 0.05 with MVA-MERS-N or MVA. Infected cells were collected at different time points after infection and titrated on CEF cells.

Recombinant virus MVA-MERS-N was formed in CEF that were infected with MVA and transfected with the MVA vector plasmid pIIIH5red-MERS-N (Figure 1a). The MVA DNA sequences in pIIIH5red-MERS-N (flank-1, flank-2) targeted the insertion of the N gene sequences into the site of deletion III within the MVA genome. The clonal isolation was facilitated by co-production of the red fluorescent reporter protein mCherry allowing for the convenient detection of MVA-MERS-N infected cells during plaque purification. The repetitive DNA sequence of flank-1 (FR) served to remove the marker gene mCherry from the genome of the final recombinant virus through initiating an intragenomic homologous recombination (marker gene deletion). After PCR analysis confirmed the presence of more than 95% MVA-MERS-N recombinant viruses in the cultures, we selected the final marker-free recombinant viruses by plaque purification and screening for plaques without mCherry fluorescence. To confirm genetic integrity and proper insertion of the heterologous N gene sequences within the MVA-MERS-N genome, we analyzed viral genomic DNA by PCR using specific oligonucleotide primers specific for MVA sequences adjacent to the deletion III insertion site (Figure 1b). Additional PCRs specific for MVA sequences within the C7L gene locus or adjacent to the major deletion sites I, II, IV, V, and VI served to control for the genetic identity and genomic stability of MVA-MERS-N (Figure 1c, and data not shown). Next, we evaluated the recombinant virus MVA-MERS-N by multi-step growth analysis in different cell lines (Figure 1d). In CEF, the cell culture routinely used to propagate recombinant MVA vaccines, MVA-MERS-N efficiently replicated to titers similar to those obtained with non-recombinant MVA. In contrast, the human cell lines HaCat and HeLa proved non-permissive for productive virus growth confirming the well-preserved replication deficiency of the recombinant MVA-MERS-N in cells of mammalian origin.

**Figure 2:**
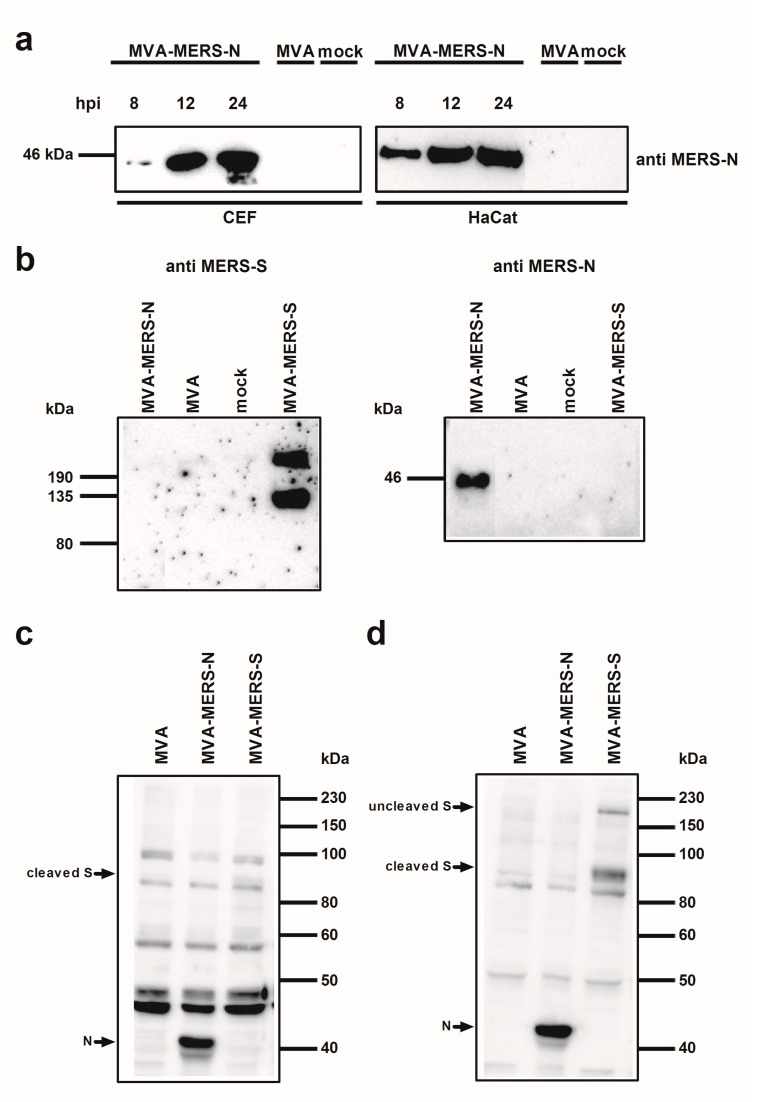
Analysis of recombinant MVA-MERS proteins; (**a**) Western Blot analysis of MERS-CoV N protein produced in CEF or HaCat cells. Lysates from cells infected with recombinant MVA (MVA-MERS-N, MVA-MERS-S) or non-recombinant MVA (MVA) at a MOI of five, or from non-infected cells (mock) were prepared at eight, 12, or 24 hpi. Proteins were analyzed by immunoblotting with a monoclonal anti-MERS-N antibody; (**b**–**d**) Western Blot analysis of MERS-CoV N and S proteins produced in CEF. Total cell extracts from CEF infected with recombinant MVA (MVA-MERS-N, MVA-MERS-S) or non-recombinant MVA (MVA) at a MOI of five, or from non-infected cells (mock) were prepared at 24 hpi. Cell lysates and proteins were tested by immunoblotting using monoclonal anti MERS-N and anti MERS-S antibody (**b**) or polyclonal sera from MERS-CoV infected rabbits (**c**) or cynomolgus macaques (**d**). Arrows indicate the N- or S-specific protein bands.

To confirm the synthesis of MERS-CoV N protein upon MVA-MERS-N infection, total cell proteins from infected CEF and HaCat cells were separated by SDS-PAGE and analyzed by immunoblotting (Figure 2). Consistent with the expected molecular mass of the MERS-CoV N protein we readily detected a ~45 kDa polypeptide using the N-specific mouse monoclonal antibody. At 24 hours post infection (hpi) a prominent band of N protein was visible in the lysates from both cell lines suggesting efficient synthesis of the recombinant protein under permissive and non-permissive growth conditions for MVA-MERS-N (Figure 2a).

In addition, the comparative Western blot analysis of cell lysates from MVA-MERS-N or MVA-MERS-S infected CEF with antigen-specific mouse monoclonal antibodies suggested the production of comparable amounts of both MERS-CoV candidate antigens (Figure 2b). This observation is in line with the fact that the MVA-MERS-S candidate vector vaccine expresses the MERS-CoV S gene sequences using the identical PmH5 promoter system [23]. As shown in previous studies, we detected two MERS-CoV S-specific protein bands upon infection with MVA-MERS-S indicating the authentic proteolytic cleavage of the full-length S glycoprotein (~210 kDa) into an N-terminal (~120 kDa S1 domain) and a C-terminal (~85 kDa S2 domain; not detected) subunit [23,50,51]. Following this, we used the total protein lysates from MVA-MERS-N or MVA-MERS-S infected CEF to assess the recognition of the MERS-CoV N and S antigens by sera from experimentally MERS-CoV infected animals. The Western blot analysis of sera from an infected rabbit (Figure 2c) or a cynomolgus monkey (Figure 2d) revealed the presence of antibodies specific for the MERS-CoV N protein. The recognition of the MERS-CoV N protein was at least as prominent as the MERS-CoV S antigen, which was suggestive of the induction of substantial N-specific antibody responses after experimental MERS-CoV infections.

### 3.2. Characterization of MERS-CoV N-specific T Cell Responses

#### 3.2.1. Initial Screen of MERS-CoV N Epitopes Using Overlapping Peptide Pools

**Figure 3:**
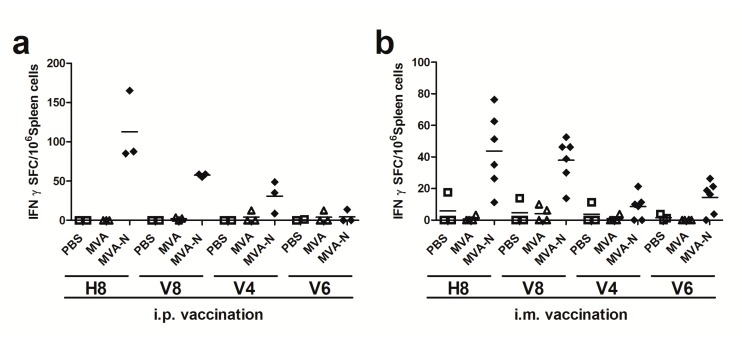
Screening for H2-d restricted T cell epitopes in MERS-CoV N protein using matrix peptide pools; (**a**–**b**) groups of BALB/c mice (*n* = 2 to 6) were vaccinated twice (21-day interval) by i.p. (**a**) or i.m. (**b**) application with 10^8^ plaque-forming-units (PFU) of recombinant MVA-MERS-N (MVA-N). Mice inoculated with non-recombinant MVA (MVA) or phosphate-buffered saline (PBS) were used as controls. Splenocytes were restimulated in vitro with pools of overlapping peptides corresponding to MERS-CoV N protein. IFN-γ spot-forming CD8+ T cells (IFN-γ SFC) were measured by ELISPOT. The lines represent means.

T cell responses against coronaviruses are known to be long lived and mostly target the more conserved CoV internal structural N protein. However, information on MERS-CoV N antigen-specific T cell specificities is still limited. Thus, we aimed first to identify N polypeptide-specific T cell epitopes in BALB/c mice immunized twice with recombinant virus MVA-MERS-N or non-recombinant MVA as a control via the intraperitoneal and intramuscular routes. Eight days after the final immunization, splenocytes were prepared and the purified CD4+ and CD8+ T cells were restimulated in vitro with overlapping peptides corresponding to the N protein. Overlapping peptides were pooled using a two-dimensional, pooled-peptide matrix system (Table S1) and screened by IFN-γ ELISPOT. The stimulation of splenoctyes from MVA-MERS-N immunized mice with the peptides from 16 out of the total 18 peptide pools did not result in the detection of IFN-γ producing T cells above background numbers obtained with splenocytes from mock or MVA-control vaccinated animals. Stimulation with the peptides from pools H8 (n = 10) and V8 (n = 10) as well as the use of the vaccinia virus positive peptide F2L [49] (data not shown) showed elevated numbers of IFN-γ spot forming cells (SFC) in CD8+ T cell cultures (Figure 3a,b). MVA-MERS-N immunizations given by i.p. and i.m. routes resulted in comparable T cell stimulatory capacities of overlapping N-specific peptides from pools V8 and H8. In contrast, peptides from other pools showed no or only minor stimulatory activities, as exemplified for peptides in pools V4 and V6. 

#### 3.2.2. Reassessment of Positive Reacting Peptides Pools for MERS-CoV N T Cell Epitopes

**Figure 4:**
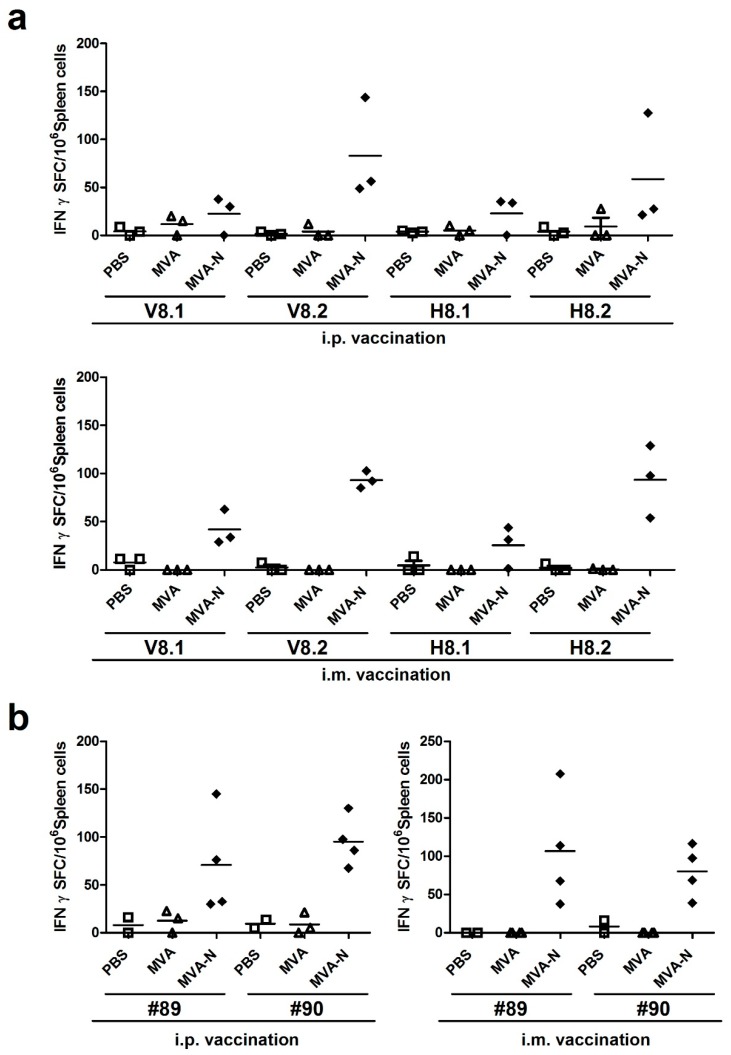
Mapping of H2-d restricted T cell epitopes in MERS-CoV N protein; (**a–b**) BALB/c mice (n = 2 to 4) were immunized twice (21-day interval) i.p. or i.m. with 10^8^ PFU of recombinant MVA-MERS-N (MVA-N), non-recombinant MVA (MVA) or PBS. Splenocytes from vaccinated mice were incubated in the presence of subpools (V8.1, V8.2, H8.1, H8.2) from positive matrix pools (**a**) or individual 15-mers peptides #89 or #90 (**b**). IFN-γ spot-forming CD8+ T cells (IFN-γ SFC) were quantified by ELISPOT. The lines represent means.

Following this, the peptides within the V8 and H8 peptide pools were used to elucidate in more detail the T cell epitope specificities. We subdivided the peptides from H8 and V8 in four new pools each containing five peptides (H8.1, H8.2, V8.1, V8.2). In addition, we separately tested the two 15-mer peptides #89 and #90, which were shared between pools H8 and V8. We again vaccinated BALB/c mice with MVA-MERS-N using i.p. or i.m. inoculations in a prime-boost regime. Splenocytes were prepared at day eight after the last vaccination and purified CD8+ T cells were restimulated with subpools V8.1., V8.2., H8.1, and H8.2 (Figure 4a). Stimulation with peptides from pools V8.1 or H8.1 activated only minor levels of IFN-γ producing cells with mean levels about 23 SFC/10^6^ splenocytes. Yet, the stimulation with subpools V8.2 and H8.2 revealed clearly higher numbers of activated T cells with 83-176 IFN-γ SFC/10^6^ splenocytes. Comparable numbers of IFN-γ producing cells were again induced by i.p. or i.m. immunization. Of note, the stimulations with the 15-mer peptides #89 (N_353-367_ = QNIDAYKTFPKKEKK) or #90 (N_357-371_ = AYKTFPKKEKKQKAP) alone resulted in detection of substantial quantities of IFN-γ producing cells (mean levels about 71–107 SFC/10^6^ splenocytes) in mice that had been vaccinated with MVA-MERS-N by both immunization routes (Figure 4b). CD8+ T cells purified from mice receiving non-recombinant MVA or mock vaccine (PBS) did not produce IFN-γ following stimulation with peptides from subpools V8.1-H8.2 and with peptides #89 and #90. When checking for the specific peptides contained within the subpools, we observed that the strongly stimulatory peptides #89 and #90 were part of the subpools V8.2 and H8.2, whereas these peptides were absent in V8.1 and H8.1. This data suggested that the overlapping 15-mer peptides #89 and #90 contained a valuable antigen epitope for the activation of MERS-CoV N-specific CD8+ T cell responses.

#### 3.2.3. Identification of MERS-CoV N-specific T Cell Epitope

To map more precisely this specific epitope within the MERS-CoV N protein, we concentrated on the overlapping 11-mer peptide shared between peptides #89 and #90 and obtained nine 8-10-mer peptides (Table 1).

**Figure 5:**
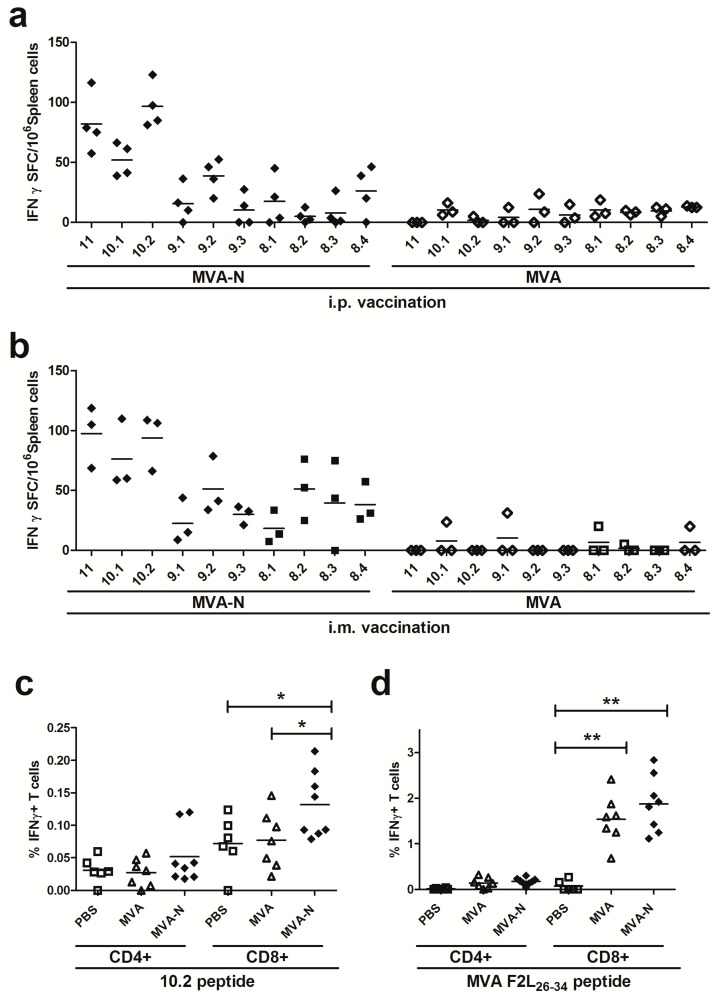
Identification of an H2-d restricted T cell epitope in MERS-CoV N protein; (**a–d**) Groups of BALB/c mice (*n* = 3 to 8) were vaccinated in a prime-boost regime with 10^8^ PFU of MVA-MERS-N via i.p. (**a**) or i.m. (**b–d**) application. Mice immunized with non-recombinant MVA (MVA) and PBS served as negative controls. (**a-b**) Splenocytes were stimulated with individual 8-11-mer peptides and IFN-γ spot-forming CD8+ T cells (IFN-γ SFC) were measured by ELISPOT. (**c–d**) Splenocytes were stimulated with positive MERS-CoV N 10.2 peptide (**c**) or F2L_26-34_ peptide (**d**) and IFN-γ producing CD8+ or CD4+ T cells were measured using intracellular cytokine staining assay and FACS analysis. The lines represent means. *< 0.05, **< 0.005.

Using these peptides for the stimulation of splenoctyes from MVA-MERS-N vaccinated mice, we obtained the highest numbers of IFN-γ producing T cells with peptide 10.2 (mean levels of 94 to 97 SFC/10^6^ splenocytes), while the other peptides (10.1, 9.1, 9.2, 9.3, 8.1, 8.2, 8.3, 8.4) induced weaker responses with a mean of 5–76 IFN-γ SFC/10^6^ splenocytes (Figure 5a,b).

Furthermore, we tested the 10.2 peptide to monitor N-specific T cell responses by IFN-γ ICS and fluorescence activated cell sorting (FACS) analysis. Indeed, we could detect significant numbers of 10.2 peptide-specific CD8+ T cells being induced and activated by the MVA-MERS-N prime-boost vaccination. In comparison, the CD4+ T cell populations from splenocytes of immunized animals demonstrated only background levels of IFN-γ producing cells (Figure 5c). The vaccinia virus-specific immunodominant CD8+ T cell determinant F2L [49] served as control peptide for the detection of MVA-specific CD8+ T cells (Figure 5d).

## 4. Discussion

The availability of appropriate MERS-CoV-specific immune monitoring tools is a prerequisite for the successful development of vaccines and therapeutic approaches. The development of these tools is hampered by the fact that we still know little about the relevant viral antigens and the overall pathogenesis of the MERS-CoV infection. Recent studies describe a number of cases with asymptomatic MERS-CoV infection in humans and raise questions as to which factors influence the clinical manifestation of MERS [52,53]. Since asymptomatic or mild clinical manifestations of MERS-CoV infection are often associated with low levels of seropositivity, the analysis of MERS-CoV-specific cellular immune responses may facilitate further insight into the immune correlates of disease prevention. Moreover, in order to characterize the pathogenesis of MERS-CoV infection, it will be indispensable to monitor the role of virus-specific T cells in animal models of MERS and to precisely identify the antigen specificities of these T cell responses.

In the present study, we identified a major histocompatibility complex (MHC) haplotype H2-d restricted peptide epitope in the MERS-CoV N protein by stimulating T cells from MVA-MERS-N vaccinated BALB/c mice with a 2-D matrix pool of overlapping peptides. These mice have already been used in various preclinical studies to establish the MERS-CoV S protein as an important vaccine antigen for induction of virus neutralizing antibodies [23,40,41,42,54]. Moreover, BALB/c mice transduced with the human cell surface receptor dipeptidyl peptidase 4 (hDPP4) using an adenovirus vector are susceptible to productive MERS-CoV lung infection, which allows for the testing of the protective efficacy of MERS-CoV-specific immunization using the MERS-CoV S protein [24,40,41,43,44]. Here, we wished to specifically assess the suitability of an MVA-delivered MERS-CoV N antigen for the activation of cellular immune responses in mice. In general, the N protein is a well conserved internal protein and the major structural component incorporating the viral RNA within the viral nucleocapsid [55]. In previous studies, the SARS-CoV N protein has also been used as candidate antigen for vaccine development [56], and an experimental DNA vaccine efficiently induced SARS-CoV N-specific cellular immunity [57,58]. In line with this data, recent studies in MERS patients demonstrated that both antibody and T cell responses are associated with recovery from MERS-CoV infection [59].

The recombinant virus MVA-MERS-N produced stable amounts of MERS-CoV N antigen upon in vitro infection of human cells indicating the unimpaired expression of the target gene at the level of viral late transcription using the synthetic vaccinia virus-specific promoter PmH5 [46]. Moreover, the MERS-CoV N antigen produced in MVA-MERS-N infected cells was strongly recognized by antibodies from experimentally infected laboratory animals suggesting that N-specific immune responses were potently activated upon MERS-CoV infection. It seems noteworthy that MERS-CoV productively replicates in rabbits, but viral loads are low and the animals develop no overt disease symptoms. However, Haagmans et al. found infectious virus in the lung tissues of the rabbits and revealed the presence of the MERS-CoV N antigen in bronchiolar epithelial cells and in the epithelial cells of the nose [60]. The localization of N in these respiratory epithelial cells may result in an efficient recognition by innate and also adaptive immune cells similar to those described for other viruses inducing robust protective immunity [61]. This might be a possible explanation for efficient activation of MERS-CoV N-specific antibodies despite a barely productive MERS-CoV infection. Similar outcomes of infection were observed upon MERS-CoV infection in cynomolgus macaques and other relevant non-human primate models [62,63]. Thus, the induction of N-specific immune responses in these animals emphasizes the potential usefulness of the MERS-CoV N protein to serve as vaccine antigen.

Nevertheless, the immunogenicity of N requires further characterization in preclinical models for MERS-CoV infection. In addition to the induction of MERS-CoV-specific antibodies, the MERS-CoV N protein holds promise to efficiently activate virus-specific CD8+ T cell responses. For more detailed studies characterizing the possible role of these T cell specificities in MERS-CoV-associated immunity or pathogenesis it is highly relevant to determine the N peptide epitopes allowing for the appropriate MHC-restricted antigen presentation and the activation of virus-specific T cells. In this study, we identified a new H2-d restricted CD8+ T cell epitope in the MERS-CoV N protein using a 2-D matrix and pools of 84 overlapping 15-mer N peptides. First, we have identified two MERS-CoV N derived peptides (N_353-367_ = QNIDAYKTFPKKEKK and N_357-371_ = AYKTFPKKEKKQKAP) and further mapped these 15-mer peptides to the minimal aa sequence of N_358-367_ = YKTFPKKEKK representing a decamer peptide epitope (Figure S1) [64]. Analysis of MERS-CoV sequences reveals that N_358-367_ is conserved among different strains of MERS-CoV (Table S2) [65]. The availability of such an epitope may allow for more detailed experimental monitoring of cellular immune responses induced by a MVA based candidate vaccine against MERS-CoV in the mouse model and potentially also in other preclinical models. Of note, the H2-d restricted CD8+ T cell epitope enables characterization of T cell responses in BALB/c mice that serve as a well-established MERS-CoV infection model following adenovirus vector mediated transduction with hDPP4 [24,40,41,43,44]. A particular feature of MERS in humans, as observed upon the investigation of cluster outbreaks in hospitals, is the lack of detectable MERS-CoV neutralizing antibodies in patients with confirmed disease [66,67]. This observation is attributed to the emergence of specific virus mutants evading the neutralizing antibody response, as already described for SARS-CoV [68,69]. Thus, future use of a T cell-specific immune monitoring might contribute to a more detailed understanding of MERS pathogenesis. Here, studies on the function of MERS-CoV-specific CD8+ T cells in this BALB/c mouse MERS-CoV lung infection model will be helpful to better estimate the role of cellular immunity in vaccine mediated protection in MERS-CoV infection.

Finally, the MVA-MERS-N vector virus generated for this study proved to be a stable recombinant virus that can be readily amplified to obtain vaccine preparations technically fulfilling all requirements for further preclinical or even clinical development. Future work with MVA-MERS-N candidate vaccines should help to elucidate the potential protective capacity of N-specific immune responses in MERS-CoV infections models and contribute to our better understanding of MERS vaccine-induced protection.

## Figures and Tables

**Table 1:** Peptide information.^1.^

Peptide-ID	Sequence	Position
#89	QNID**AYKTFPKKEKK**	N_353-367_
#90	**AYKTFPKKEKK**QKAP	N_357-371_
11	AYKTFPKKEKK	N_357-367_
10.1	AYKTFPKKEK	N_357-366_
10.2	YKTFPKKEKK	N_358-367_
9.1	AYKTFPKKE	N_357-365_
9.2	YKTFPKKEK	N_358-366_
9.3	KTFPKKEKK	N_359-367_
8.1	AYKTFPKK	N_357-364_
8.2	YKTFPKKE	N_358-365_
8.3	KTFPKKEK	N_359-366_
8.4	TFPKKEKK	N_360-367_

^1^ The common 11 amino acid sequence between positive 15-mers #89 and #90 was truncated into 8-10-mer peptides and tested by ELISPOT and ICS assay.

## Supplementary Materials

The following are available online at http://www.mdpi.com/1999-4915/10/12/718/s1, Table S1: The design of matrix peptide pools for systematically screening of H2-d restricted T cell epitopes in MERS-CoV N protein protein, Figure S1: Sequence analysis and modular organization of MERS-CoV N protein, Table S2: Comparative analysis of MERS-CoV N_358-367_ epitope in different MERS-CoV strains.
