# Supplementary material for: CD8+ T Cells Responding to the Middle East Respiratory Syndrome Coronavirus Nucleocapsid Protein Delivered by Vaccinia Virus MVA in Mice
Source: Viruses. 2018 Dec 16;10(12):718. doi: 10.3390/v10120718 (PMC6316859; doi:10.3390/v10120718)
Supplement: Supplementary file 1 [file viruses-10-00718-s001.pdf]

viruses
MDPI

Supplementary Materials:

Table S1. The design of matrix peptide pools for systematically screening of H2-d restricted T cell epitopes in MERS-CoV N protein.

Matrix	Pool	Peptide								
1	H1	2	3	4	6	7	8	9	10	11
	H2	12	13	14	15	16	17	18	19	22
	H3	23	24	25	26	27	28	29	30	31
	H4	32	33	34	35	36	37	38	39	49
	H5	50	51	52	53	54	55	56	57	58
	H6	59	60	65	66	67	68	69	70	71
	H7	72	73	74	75	76	77	78/79	80	81
	H8	82	83	84	85	86	87	88	89/90	91
	H9	92	93	94	95	96	97	98	99	100/101
2	V1	2	12	23	32	50	59	72	82	92
	V2	3	13	24	33	51	60	73	83	93
	V3	4	14	25	34	52	65	74	84	94
	V4	6	15	26	35	53	66	75	85	95
	V5	7	16	27	36	54	67	76	86	96
	V6	8	17	28	37	55	68	77	87	97
	V7	9	18	29	38	56	69	78/79	88	98
	V8	10	19	30	39	57	70	80	89/90	99
	V9	11	22	31	49	58	71	81	91	100/101

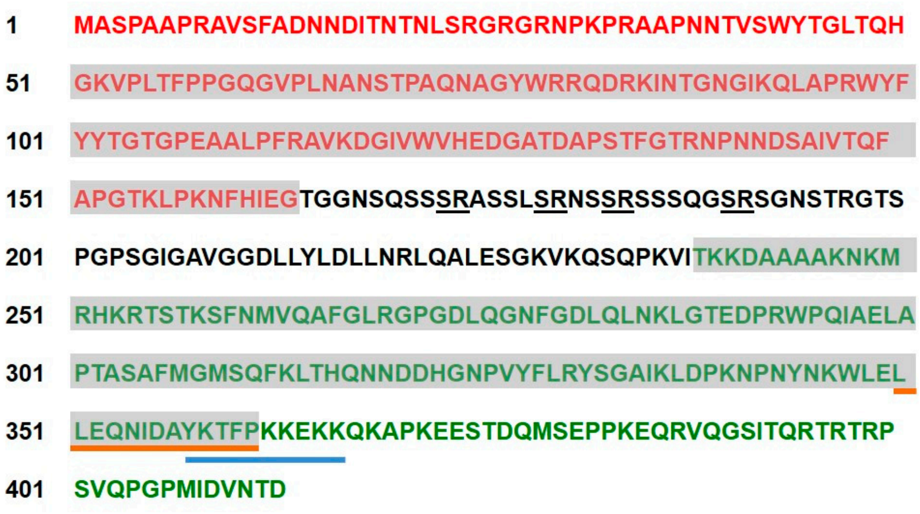

Figure S1. Sequence analysis and modular organization of MERS-CoV N protein. The primary amino acid sequence of MERS-CoV N protein (MERS-CoV strain EMC/2012, GenBank accession no. JX869059). The N-terminal and C-terminal domain are indicated in red and green, respectively. Predicted RNA-binding domains (aa 37-164 and aa 239-362) are highlighted in gray. The structurally flexible linker region is indicated in black containing several Ser/Arg (SR) motifs, which are underlined in black [64]. H2-d restricted peptides with CD4+ ($N_{350\text{-}362}$, [57]) and CD8+ ($N_{358\text{-}367}$) T cell antigenicity are underlined in orange and blue, respectively.

Table S2. Comparative analysis of MERS-CoV $N_{358-367}$ epitope in different MERS-CoV strains (modified from [65]).

Lineage	Strain	$N_{358-367}$
Lineage 1	England-Qatar/2012	YKTFPKKEKK
Lineage 2	Abu Dhabi_UAE_8_2014	YKTFPKKEKK
Lineage 3	Riyadh_2014KSA_683/KSA/2014	YKTFPKKEKK
Lineage 4	Jeddah_c7770/KSA/2914-04-07	YKTFPKKEKK
Lineage 5	MERS-CoV/KOR/KNIH/0001_05_2015	YKTFPKKEKK
Outgroup	MERS-CoV EMC/2012	YKTFPKKEKK

© 2018 by the authors. Submitted for possible open access publication under the terms and conditions of the Creative Commons Attribution (CC BY) license (http://creativecommons.org/licenses/by/4.0/).
